# Development of Solid SEDDS, V: Compaction and Drug Release Properties of Tablets Prepared by Adsorbing Lipid-Based Formulations onto Neusilin® US2

**DOI:** 10.1007/s11095-013-1106-4

**Published:** 2013-06-25

**Authors:** Suhas G. Gumaste, Damon M. Dalrymple, Abu T. M. Serajuddin

**Affiliations:** 1 Department of Pharmaceutical Sciences, College of Pharmacy and Health Sciences, St. John’s University, 8000 Utopia Parkway, Queens, NY 11439 USA; 2 ABITEC Corporation, 501 W. 1st Avenue, Columbus, OH 43215 USA

**Keywords:** SEDDS, Porous silicate, Neusilin®US2, Tablets, Powder properties, Tensile strength, Dispersion test, Gel formation

## Abstract

**Purpose:**

To develop tablet formulations by adsorbing liquid self-emulsifying drug delivery systems (SEDDS) onto Neusilin®US2, a porous silicate.

**Methods:**

Nine SEDDS were prepared by combining a medium chain monoglyceride, Capmul MCM EP, a medium chain triglyceride, Captex 355 EP/NF, or their mixtures with a surfactant Cremophor EL, and a model drug, probucol, was then dissolved. The solutions were directly adsorbed onto Neusilin®US2 at 1:1 w/w ratio. Content uniformity, bulk and tap density, compressibility index, Hausner ratio and angle of repose of the powders formed were determined. The powders were then compressed into tablets. The dispersion of SEDDS from tablets was studied in 250 mL of 0.01NHCl (USP dissolution apparatus; 50 RPM; 37°C) and compared with that of liquid SEDDS.

**Results:**

After adsorption of liquid SEDDS onto Neusilin®US2, all powders demonstrated acceptable flow properties and content uniformity for development into tablet. Tablets with good tensile strength (>1 MPa) at the compression pressure of 45 to 135 MPa were obtained. Complete drug release from tablets was observed if the SEDDS did not form gels in contact with water; the gel formation clogged pores of the silicate and trapped the liquid inside pores.

**Conclusion:**

Liquid SEDDS were successfully developed into tablets by adsorbing them onto Neusilin®US2. Complete drug release from tablets could be obtained.

**Electronic supplementary material:**

The online version of this article (doi:10.1007/s11095-013-1106-4) contains supplementary material, which is available to authorized users.

## INTRODUCTION

The lipid-based delivery system is a highly promising approach to enhance bioavailability of poorly water-soluble compounds since it presents the drug to the gastro-intestinal tract in a solubilized state. There are numerous reports in the literature describing the advantages of lipid-based systems, such as self-emulsifying drug delivery systems (SEDDS), self-microemulsifying drug delivery systems (SMEDDS), self-nanoemulsifying drug delivery systems (SNEDDS), etc., for the formulation of poorly water-soluble drugs. However, the commercial application of the technology in marketed products is still limited as there are only few major lipid-based products available (1).

One of the primary reasons for the lack of widespread application of lipid-based formulations is that the most commonly used lipids and surfactants are liquid and, thus, not amenable to the development of solid dosage forms. They usually lead to liquid-filled bottles (Agenerase®, GSK; Sustiva Oral Solution, BMS) and, if the solubility of drug is high enough or the dose is low, to liquid-filled soft or hard gelatin capsule formulations (Avodart®, GSK; Aptivus®, Boehringer Ingelheim; Glakay® Capsules and Juvela®N Soft Capsules, Eisai) (2). Such formulations, however, have many limitations and challenges. First, oral solutions have limited patient acceptance. Second, if the solution is encapsulated in soft gelatin capsules, the drug loading could be limited by the fill weight and the drug solubility (3–5), and there is a potential risk of drug precipitation if the soft gelatin capsules are not well-formulated (6). Since most of the major pharmaceutical companies do not have soft gelatin encapsulation facilities, it might also be necessary to outsource the development and the manufacture of soft gelatin capsules.

To obviate the need for developing solution formulations, lipid-based semi-solid formulations that may be filled into hard gelatin capsules have been developed (7–10). However, the understanding of physical stability, polymorphism and phase changes of such systems is still challenging (6,11,12). Also, there are some unique manufacturing concerns with the development of semisolid-filled hard gelatin capsules, including heating required during manufacturing and filling of semi-solids, vulnerability to ‘stringing’ of matrix in filling-machine nozzles, and the need for sealing or coating of hard gelatin capsules to prevent any potential leaking (13).

To address the above-mentioned issues with liquid and semi-solid formulations, various studies were conducted to solidify SEDDS into dry powders (14–20). Usually, liquid SEDDS were adsorbed onto porous carriers like silicates to obtain dry powders. Since the powder formulations are primarily designed for filling into hard gelatin capsules, there is a limitation of how much powders may be filled into a capsule, especially by considering that most of the silicates have low bulk densities. This limits the drug load per unit dose. Tablet is apparently a better alternative than both soft and hard gelatin capsules for delivering SEDDS as solid dosage forms. It has better patient acceptance and more economical manufacturing process than soft gelatin capsules, and it may have better physical and chemical stability than liquid and semisolid-filled soft and hard gelatin capsules. A tablet can also have a higher drug load than a hard gelatin capsule, because 2 to 3 times more powders can usually be compressed into tablet than what can be filled in a hard gelatin capsule. Despite the obvious advantages of tablets, there are very few reported studies on the development of tablet formulations for SEDDS. One of the major reasons for this situation could be the difficulty in finding the right carrier that can carry adequate lipid load and at the same time exhibit good powder flow properties and tabletability. When powders were compressed into tablets, they usually exhibited low tablet hardness due to low compressibility of silicates and the ‘squeezing out’ of adsorbed liquids from the silicates under compression (21). Tablet formulations of lipid-based drug delivery systems exhibit high disintegration time due to the hydrophobic environment within tablets created by the presence of lipids (22–24). Tablets also exhibit incomplete drug release, which has been attributed to irreversible interaction between the self-emulsifying components and the carrier (22). Particle size, surface area and the pore length of carriers were also reported to be responsible for incomplete drug release (21,23).

The review of literature thus reveals that there is no practical approach of developing tablet formulations for SEDDS that would have adequate liquid load, acceptable tabletability and complete drug release. The objective of the present investigation has, therefore, been to develop strategies to overcome some of the challenges facing the development of tablet formulations for SEDDS. In a previous study, we studied six commercially available silicates for their ability to adsorb lipids and surfactants and form compacts (25). Among those silicates, only Neusilin® US2 exhibited acceptable tabletability when lipid and surfactant were adsorbed onto it at the solid to liquid ratio of 1:1 w/w. Neusilin® US2 was, therefore, used in the present investigation to develop a process for loading lipid-based formulations onto the carrier, and the powders produced were then tested for flow properties and tabletability. Since the incomplete release of drug was reported to be a major issue for such formulations (18,20,21,23,26,27), effects of composition and physicochemical properties of adsorbed liquids on drug release from tablets were studied, and formulations that would lead to complete drug release were identified. Probucol, which is a neutral, virtually non-ionizable compound, with an extremely low solubility of 2–5 ng/mL and a log P value of 11 (28,29), was used as the model drug.

## MATERIALS AND METHODS

### Materials

Capmul MCM EP (glycerol monocaprylocaprate) and Captex 355 (caprylic/capric triglycerides) were supplied by ABITEC Corp., Columbus, OH, USA, and Cremophor EL (PEG-35 castor oil) was donated by BASF, Tarrytown, NY, USA. Chemical structures and compositions of these components were described earlier (30). The model drug probucol was purchased from Sigma Aldrich, St. Louis, MO, USA. Neusilin® US2 was supplied by Fuji Health Science, Burlington, NJ, USA. Croscarmellose sodium (Vivasol® GF), which was used as the disintegrant in tablets, was obtained from JRS Pharma, Rosenberg, Germany. All other reagents and chemicals used were of analytical grade or better.

### Preparation of Liquid SEDDS

Compositions of various liquid SEDDS prepared for subsequent conversion into solid dosage forms by adsorption onto the silicate powder are given in Table I. The compositions representing different medium chain lipids (monoglyceride *versus* triglyceride) and their mixtures, different lipid to surfactant ratios, and varied performance upon dilution with water (particle size, gel formation, etc.) were selected based on phase diagrams constructed earlier in our laboratory (30). They were prepared by mixing glycerol monocaprylate (Capmul MCM EP), caprylic/capric tricaprylate (Captex 355 EP/NF) and PEG-35 castor oil (Chremophor EL) in different proportions. Formulations F1, F2 and F3 were, respectively, 7:3, 1:1 and 3:7 w/w mixtures of the lipid Capmul MCM EP (glycerol monocaprylocaprate) and the surfactant Cremophor EL (PEG-35 castor oil). Upon dilution with water (1:99 w/w), the formulations form fine emulsions with averages particle sizes of 820, 380 and 280 nm, respectively, and there was no gel formation along their dilution paths (30). Formulations F4, F5 and F6 were, respectively, mixtures of Captex 355 EP/NF (caprylic/capric triglyceride) and Cremophor EL, and they form fine emulsions or microemulsions of, respectively, 270, 180 and 160 nm particle sizes upon dilution with water (1:99 w/w). The major difference between the use of the monoglyceride (Formulations F1, F2 and F3) and the triglyceride (Formulations F4, F5 and F6) was that the monoglyceride-surfactant mixtures converted to fine emulsions upon dilution with water without undergoing any gel formation in the process. In contrast, the triglyceride-surfactant mixtures initially formed gels upon dilution with water, which then converted to fine emulsions or microemulsions. Similar lipid to surfactant ratios were also used for Formulations F7, F8 and F9. However, instead of using a single lipid component as in F1 to F6, the 1:1 w/w mixture of monoglyceride (Capmul MCM EP) and triglyceride (Captex 355 EP/NF) was used as the lipid in preparing Formulations F7, F8 and F9. The use of the combination of lipids in preparing SEDDS results in the formation of microemulsions of 40, 24 and 19 nm particle size, respectively, without undergoing any gel formation along the dilution paths with water (30). Formulations F10, F11, F12 and F13 were for individual surfactant, lipids or the mixture of lipids, and *per se* they are not SEDDS; the primary purpose of their use was to investigate the impact of individual components on the tableting properties of Neusilin® US2. The model drug probucol was dissolved in different liquids such that the concentrations were kept at 80% of saturation solubility.

**Table I:** Compositions of Liquid Formulations Used for Adsorption onto Neusilin®US2

Formulation Code	Capmul MCM (% of total liquid mixture)	Captex 355 (% of total liquid mixture)	Cremophor EL (% of total liquid mixture)	Drug (probucol) load mg/g of mixture^a^
F1	70	0	30	62
F2	50	0	50	59
F3	30	0	70	61
F4	0	70	30	107
F5	0	50	50	123
F6	0	30	70	78
F7	35	35	30	93
F8	25	25	50	92
F9	15	15	70	87
F10	0	0	100	49
F11	100	0	0	42
F12	0	100	0	106
F13	50	50	0	97

^a^Saturation solubility values of probucol used in calculating the drug load were generated in our laboratory by Ms. H. N. Prajapati

### Adsorption of Liquid SEDDS onto Silicate

Neusilin® US2 was used as the silicate of choice based on a previous study where the silicate retained acceptable tableting properties after incorporation of lipids and surfactants (25). Although an organic solvent was used to adsorb lipids onto silicates in the previous study, attempts were made in the present investigation to develop a solvent-free method for adsorption of liquid SEDDS onto Neusilin® US2. A lab scale mixing assembly was set using a twisted blade stirrer (Model 5 VB-RS, Blade diameter 75 mm, Eastern Mixers, Clinton, CT, USA) and a 500-mL glass beaker. The beaker was selected such that it would allow a very narrow clearance between the stirrer blade and the beaker wall to avoid material build up at the periphery. A typical batch size of the formulation was ~50 g containing 25 g of liquid with composition as per Table I, 25 g of Neusilin® US2 and the required amount of probucol dissolved in the liquid phase. The weighed amount of Neusilin® US2 was added gradually to the weighed amount of the solution in the beaker at a constant stirrer speed 550 RPM. The addition of the adsorbent powder was completed in 1 min and the mixing was then continued for an additional 4 min with intermittent pausing to release lumps from the edges of the container by scraping them with a spatula. A free flowing powder was obtained at the end of the mixing process. To conserve materials, the test for the content uniformity of drug in formulations was conducted with batch sizes of 30 g.

SEM images were recorded for powders prepared by the solvent-free method, and they were then compared with the SEM images of powders prepared by using the solvent method. The SEM images of Cremophor EL and Captex 355 EP/NF adsorbed onto Neusilin® US2 at 1:1 w/w ratio are submitted with this paper as Supplementary Material, which demonstrate that there was no difference in morphology and surface structures of the powders obtained by the solvent or the solvent-free methods. The solvent-free method was, therefore, adopted for all studies in the present investigation.

### Characterization of Powder Properties

Although there were no visible lumps after adsorption of liquids onto Neusilin® US2, the formulations were passed through an 800 μm sieve before further processing to ensure the absence of any large aggregates. The flow characteristics of various formulations were then determined by measuring the angle of repose, Carr’s compressibility index, and the Hausner ratio as per the United States Pharmacopoeia (31). To determine the angle of repose, 25 g of powder was poured through a glass funnel with a bore diameter of 11.4 mm and situated 4 in. from the base. The diameter of the base and the height of the powder cone formed were measured to determine the angle of repose. For determining the compressibility index and the Hausner ratio, 15 g of a powder (5 g in case of neat Neusiln® US2) was poured into a 50-mL measuring cylinder, the bulk and the tapped densities of the powder were measured, and the density values obtained were then utilized to calculate compressibility index and Hausner ratio.

### Tablet Compression and Characterization

To identify the optimal tablet compaction pressure, tablets, with the weight of *ca*. 800 mg each, were compressed at different pressures in the range 20 MPa to 230 MPa using 14 mm flat face punches (Natoli Engineering, Saint Charles, MO) on a single punch Carver Press assembly (Carver Inc., Wabash, IN). Tablet hardness was determined using a PAH-01 hardness tester (Pharma Alliance Group, Valencia, CA), and the tensile strengths (*ρ*) of the tablets were then calculated using the following equation:

$$ \rho =2F/\pi DT $$

where F is the breaking force, and D and T are, respectively, the diameter and thickness (32). For friability testing, separate tablets, weighing *ca*. 540 mg each, were compressed using 11.6 mm flat faced punches (Natoli Engineering, Saint Charles, MO) at 135 MPa. The test was conducted using an Erweka TA 10 friability tester (Erweka America Corp., Annandale, NJ) at 100 rotations by using 5 tablets each time, and the tablets were weighed before and after rotations. Tablets for the determination of tensile strength and the friability testing were compressed without incorporating any disintegrant to avoid its interference in the inherent tabletability of the silicate before and after adsorption of liquids. It was expected that the presence of a limited amount of polymeric disintegrants might have only a minor effect on tabletability and friability of the formulations. If at all, the effect would be positive due the plastic nature of the disintegrant upon compression.

### Dispersion Test

#### Liquid SEDDS

Liquid formulations prepared according to Table I were filled in size 00 hard gelatin capsules (~500 mg/capsule) for testing their dispersion in 250 mL of 0.01 N HCl (pH ~ 2) at 37°C according the procedure described earlier (30,33). The USP Type II dissolution apparatus was used at 50 RPM. Aliquots of solution (3 mL each) were withdrawn from dispersion vessels at 15, 30, 60, 45, 120 and 180 min and assayed for the globule size of dispersed lipids (Delsa Nano C particle size analyzer, Beckman Coulter Inc., Brea, CA) and the drug content. The aliquots were not filtered before analysis as the dispersed lipids were partially retained on filters during the process. For analysis of drug concentration by HPLC, the unfiltered aliquots were diluted with methanol to dissolve the dispersed lipids. The HPLC conditions for analysis were: C8 reverse phase column (3.5 μm × 4.6 mm × 150 mm); methanol/water (95:5 v/v) mobile phase 1 mL/min flow rate; and λ = 243 nm UV detection wavelength. The dispersion of liquid SEDDS served as the control for the dispersion test of tablet formulations.

#### Tablets

Tablets weighing *ca*. 840 mg (~765 mg of liquid and drug loaded silicate plus ~10% disintegrant) were compressed at 145 MPa and were dispersed according to the procedure described above for liquid SEDDS. Aliquots of dissolution media (3 mL each) were collected at 15, 30, 60, 120 and 180 min using a 5-mL syringe fitted with a relatively coarse filter of 5 μm pore size such that only the Neusilin US2 particles but not the lipid globules were retained on filters. The same filter was attached to another 5-mL syringe to push back 3 mL of fresh dissolution medium to replace the aliquot withdrawn from the dissolution vessel. This helped to restore and resuspend the silicate particles entrapped on the filter during sampling back into the dissolution medium. At the end of 3 h of dispersion testing, the entire content of the dissolution vessel was passed through a filter with 25 μm pore size to separate the silicate from the dissolution medium. The filtrate, the residue on the filter and any silicate particles sticking with the inner surface of the vessel were analyzed separately for drug content. The filtered medium was also analyzed for the particle size of the dispersed lipids. To determine the effect, if any, of compression on the rate and amount of drug released the experiments were repeated without compression, where the formulation was the same but instead of compression, the powders were weighed and added directly to the dispersion vessel.

## RESULTS AND DISCUSSION

Tabletability, which is characterized by the plot of tablet tensile strength as a function of compaction pressure, may be defined as the capacity of a powder to be transformed into a tablet of specified strength under the effect of compaction pressure (34). As reported earlier, neat silicates, especially those commonly used in pharmaceutical products, exhibit very low bulk densities and lack inherent tabletability (25). There is a significant improvement in density of the silicates after loading of liquid lipid-based formulations. However, the tabletability does not increase with the addition of liquid, and in most cases it decreases. Several investigators incorporated large amounts of binders and diluents to increase tabletability of silicate formulations (22–24). This approach may often defeat the purpose of solidifying the lipid-based formulations as the added excipients increase the tablet size and, therefore, only limited amount of the liquid SEDDS can be incorporated in a tablet of convenient size. For these reasons, Neusilin® US2 was used in the present investigation without the addition of any other excipients, except for a disintegrant.

### Powder Properties

#### Flow Properties

Direct incorporation of lipids, surfactants or their mixtures with Neusilin® US2 at 1:1 w/w ratio using a solvent-free method resulted in dry powders. The appearance of the powders was such that one would have difficulty to recognize visually that the adsorbent contained an equal weight of oily liquid. However, it is essential that the powders have good flow for their successful development as tablets (34), especially if the powders are intended to be compressed using the high-speed tablet press (35). Well-established compendial methods of angle of repose, Carr’s compressibility index and Hausner ratio were used to characterize flow properties of different formulations (31). Table II shows the flow properties as well as bulk and tap densities of all formulations listed in Table I after their adsorption onto Neusilin® US2 at 1:1 w/w. The same properties of neat Neusilin® US2 are also given for comparison. To establish the reproducibility of the methods, the effect of individual lipid-based formulation components Cremophor EL, Capmul MCM EP and Captex 355 EP/NF (F10, F11 and F12, respectively) on densities and flow properties of Neusilin® US2 were first tested in triplicate. The results were highly reproducible with very low standard deviations. Since the physical properties of Formulations F1 to F9 and F13 were also found to be similar, tests for those formulations were conducted one time only. A marked improvement in the bulk and tapped densities of Neusilin® US2 was observed upon loading each of the formulations at 1:1 w/w ratio as compared to those of the neat silicate. The bulk and tap densities of the formulations were 0.34–0.37 and 0.41–0.43 g/cc, respectively, as compared to 0.17 and 0.19 g/cc, respectively, for the neat silicate. Thus, the density of Neusilin® US2 approximately doubled after loading of the liquids. Since the liquid formulations were added to the silicate at 1:1 w/w ratio, the doubling of the density of the powders, however, appears to be due to the adsorption of liquids into the pores (same volume, double the weight) without changing other physical properties of the silicate.

**Table II:** Density and Flow Properties of Powders After Adsorption of Liquid Formulations onto Neusilin® US2 at 1:1 w/w ratio

Name/Formulation Code	Density (g/cc)		Compressibilty Index^a^		Hausner Ratio^a^		Angle of Repose	
	Bulk	Tap	Value	Classification	Value	Classification	Value	Classification
Neusilin US2 (neat)	0.17	0.19	13	Good	1.15	Good	22	Excellent
F1	0.34	0.41	17	Fair	1.20	Fair	18	Excellent
F2	0.35	0.41	15	Fair	1.17	Good	20	Excellent
F3	0.37	0.42	13	Good	1.15	Good	19	Excellent
F4	0.35	0.41	17	Fair	1.20	Fair	21	Excellent
F5	0.37	0.43	14	Good	1.16	Good	21	Excellent
F6	0.38	0.43	14	Good	1.16	Good	18	Excellent
F7	0.37	0.42	12	Good	1.14	Good	19	Excellent
F8	0.38	0.42	11	Good	1.13	Good	18	Excellent
F9	0.36	0.42	14	Good	1.16	Good	19	Excellent
F10^b^ (Cremophor)	0.35 (0.00)	0.41 (0.00)	14 (0.67)	Good	1.17 (0.01)	Good	19 (0.10)	Excellent
F11^b^ (Capmul)	0.36 (0.01)	0.42 (0.02)	14 (1.71)	Good	1.16 (0.02)	Good	20 (0.92)	Excellent
F12^b^ (Captex)	0.37 (0.01)	0.43 (0.01)	15 (0.70)	Fair	1.18 (0.01)	Good	19 (1.20)	Excellent
F13	0.37	0.43	14	Good	1.16	Good	19	Excellent

^a^As per the United States Pharmacopoeial method (31)

^b^ n = 3 (± S.D.)

The Compressibility Index (CI), which is also known as the Carr’s Index, and the Hausner Ratio (HR), which is essentially the ratio of the bulk density to the tap density of powders, are interrelated according to HR = 100/(100 - CI), and they usually reflect how easily the arch formed by powders on the hopper of a tablet press could be broken. The values of these two indices are indirectly influenced by bulk density, tap density, size, shape, moisture content and cohesiveness of the materials and, therefore, serve as useful tools to assess powder properties. In the present investigation, the CI and HR values of all formulated powders were mostly ‘good’ and in a few cases ‘fair’ according to the USP classification system (31), indicating that there would not be issues with the flow of powders from hoppers. The angle of repose is an old and simple technique that also gives a general idea of the cohesivity and flow properties of powder; however, it is heavily affected by the methodology of the test and may not be highly reproducible (36). Nonetheless, neat Neusilin® US2 and all formulated powders exhibited ‘excellent’ angles of repose as per the USP guideline.

The good flow properties of Neusilin® US2 both before and adsorption liquid SEDDS could be explained by analyzing the shape, size and porosity of its particles. It was observed by scanning electron microscopic studies that Neusilin® US2 particles are spherical and free from rough edges (25). Such particles tend to flow freely since they avoid inter-particulate entanglement caused by irregularly shaped particles and the ones with rough edges. Also, as reported earlier, the particles of Neusilin® US2 are relatively large (60–120 μm) and highly porous, and, as a result, the adsorbed liquids are localized or lodged deep inside the pores of the spherical particles, leaving very little liquids on the surface of particles (25). Thus, there would also be little or reduced chance of particle bridging induced by inter-particulate liquids. A similar mechanism was proposed by Agarwal *et al*. (18), who studied the adsorption of lipids by several silicates at different silicate to oil ratios and observed that the flow properties of most of the silicates improved after initial loading of liquids (ball-bearing effect), then remained constant with increasing liquid to silicate ratios (localization inside the silicate particles), and finally deteriorated at very high ratios of liquid to silicates (liquids covering the surface).

#### Content Uniformity

**Fig. 1:**
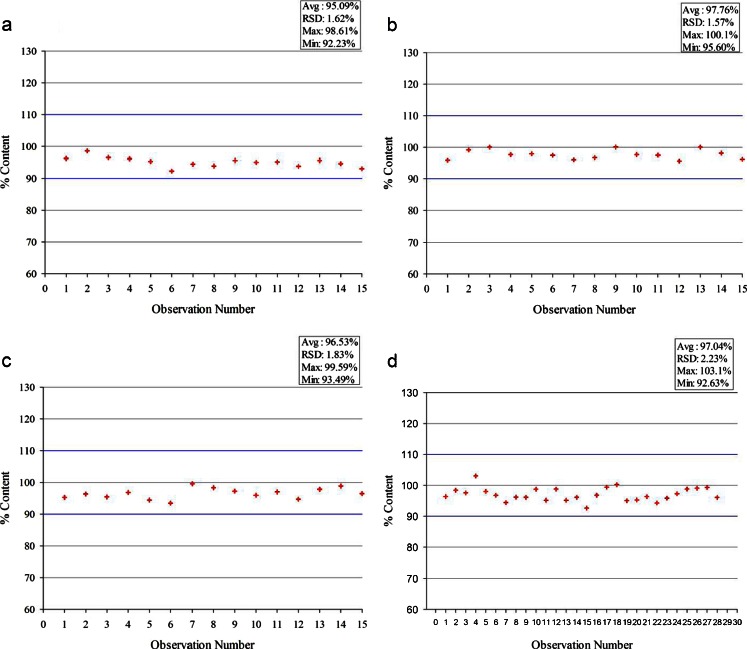
Drug content uniformity test results for powders prepared by adsorbing (**a**) PEG-35 castor oil (Cremophor EL) (F10), (**b**) glycerol monocaprylocaprate (Capmul MCM EP) (F11), (**c**) caprylic/capric triglyceride (Captex 355 EP/NF) (F12) and (**d**) the rest of the formulations (F1-F9 & F13) onto Neusilin® US2.

To determine whether the liquid SEDDS may be distributed uniformly within the silicate bed, the content uniformity of the drug was studied after adsorption of Formulations F10, F11 and F12 (drug solutions in neat Cremophor EL, Capmul MCM EP and Captex 355 EP/NF, respectively) in triplicate. Figure 1a, b and c show the content uniformity data for F10, F11 and F12, respectively; there are 15 data points in each figure as three batches of formulation were prepared and 5 samples (1 g each) were collected from each batch. Figure 1d shows the cumulative content uniformity results for the rest of the formulations (F1-F9 and F13), where 3 samples were collected from each batch, giving a total of 30 data points. The formulations exhibited RSD < 3%, and the average drug content in the samples was around 96.5%. According to the US FDA draft guidance for ‘blend uniformity’ (37), a batch would ‘readily pass’ if the assay values 60 or more samples from a batch have RSD ≤ 4.0%. Although the collection of such a large number of samples was not possible at the lab scale, the results indicate that the acceptable content uniformity may be obtained by the process developed in the present investigation. Less than the expected 100% drug content in the powders could possibly be due to sticking of the liquids to the container wall during the processing of different formulations. It is expected that the loss of drug would decrease if the batch size is increased.

### Tablet Properties

#### Selection of Compression Pressure

**Fig. 2:**
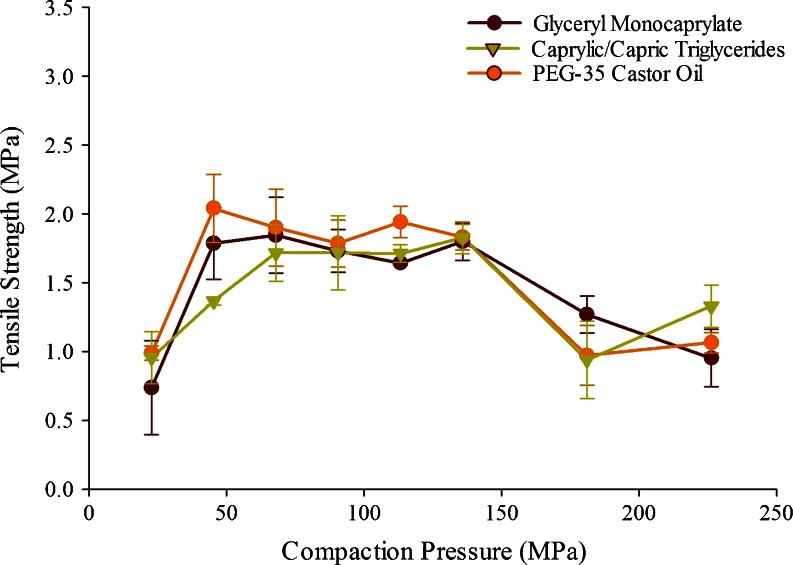
Comparative evaluation of tabletability of Neusilin® US2 at 1:1 ratio with PEG-35 Castor oil (Cremophor EL) (F10); glycerol monocaprylocaprate (Capmul MCM EP) (F11); and caprylic/capric triglyceride (Captex 355 EP/NF) (F12) (*n* = 3).

Tensile strength values in excess of 1 MPa are typically desired for tablets to withstand stress during their lifetime (38). It was observed earlier that tablets with acceptable tensile strength (>1 MPa) could be prepared for 1:1 w/w lipid-Neusilin® US2 mixtures at compression pressures ranging from 45 to 135 MPa (25). However, the formulations were prepared in the previous study by first dissolving the lipids in an organic solvent. To confirm the results for the solvent-free method adopted in the present investigation, tabletability studies were conducted for Formulations F10, F11 and F12 in triplicate, and the results are shown in Fig. 2. It is evident from Fig. 2 that the tensile strengths of all the three formulations are essentially similar throughout the range of the pressures employed. It also appears that there could be a plateau in tensile strength of the tablets between 45 and 135 MPa, and subsequently the tensile strength appeared to decrease. The relative insensitivity of tensile strength to pressure from 45 to 135 MPa, could be attributed to interplay between the liquid spreading throughout the tablet (partially inhibiting bonding between carrier particles) and new bonds being formed between the carrier particles (due to fracture of the carrier material with increasing pressure) (39). The decrease in tensile strength at higher pressures (>135 MPa) may be attributed to the squeezing out of the liquid from the pores of Neusilin® US2, thus interfering in the bonding between the silicate particles.

Since the acceptable compaction pressure ranges from 45 to 135 MPa, ideally one would pick the least possible compaction pressure for tableting. This would allow maximum porosity within the tablet, minimize disintegration time and the amount of disintegrant required, minimize internal stresses, and reduce the extent of elastic recovery, if any. In this respect, the compaction pressure of 45 MPa would appear optimal. Although the tablets produced at 45 MPa exhibited excellent appearance and tensile strengths, the friability test revealed that there was slight chipping of tablets at the edges. Tablets produced at increasing compaction pressures (ca. 70, 90, 115, 135 MPa) had progressively decreased chipping. Since 135 MPa was the maximum pressure that could be used without compromising the tensile strength, it was utilized for further studies. Flat faced tablets are known for chipping at the edges during ejection from dies as well as during friability testing and coating. It is expected that the slight chipping of tablets observed at135 MPa may be eliminated by using tablets without sharp edges, such as, convex or beveled edge tablets.

Further, to assess any possible change in tabletability due to stress relaxation of the tablets, Formulations using F10 (neat Cremophor EL), F11 (neat Capmul MCM EP) and F12 (neat Captex 355 EP/NF) were compressed at 135 MPa and tested for tablet thickness, diameter and hardness at 24 h and 15 days. No significant change in any of these measurements with time was observed, indicating that the compression process was a robust one.

#### Friability Testing

**Fig. 3:**
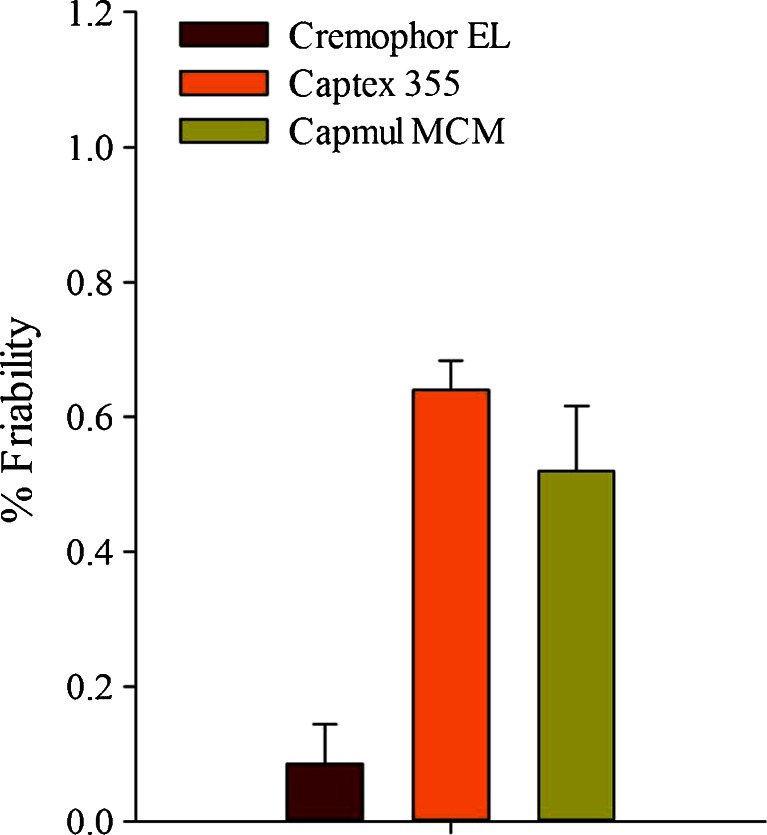
Comparative friability of tablets prepared with Cremophor EL (F10), Capmul MCM EP (F11) and Captex 355 EP/NF (F12) compressed at 135 MPa (sample size = 5 tablets).

Figure 3 shows the friability of the tablets compressed at 135 MPa using 11.6 mm flat face punches. Noticeably the tablets obtained using Cremophor EL exhibited less friability (0.1%) than those using Capmul MCM EP (0.52%) and Captex 355 EP/NF (0.64%). Although all values are low, the relatively lower friability with Cremophor EL could possibly be related to its higher viscosity as compared to the other two liquids. With an adequate tensile strength to the tablets, it appeared that tablet chipping was the major contributor to the tablet friability.

#### Optimization of Disintegrant Level

**Fig. 4:**
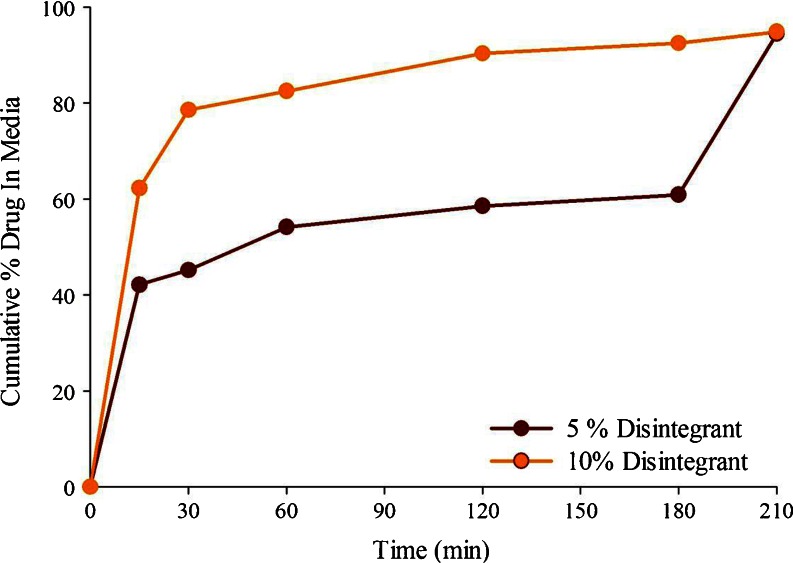
Effect of disintegrant (croscarmellose sodium) level on the dispersion of the liquid lipid-based formulation (F3) loaded onto Neusilin® US2 in 250 mL of 0.01 N HCL at 37°C and 50 RPM. For tablets with 5% disintegrant, the paddle rotation was changed to 250 RPM after 180 min.

Due to the hydrophobic microenvironment of tablets containing lipids, they failed to disintegrate fully in absence of a disintegrant. Croscarmellose sodium (cross-linked carboxymethyl cellulose), which is considered to be a superdisintegrant because of its efficacy at a relatively low concentration in tablet (~2%) (40), was, therefore, used as the disintegrant. The target maximum disintegration time in the present study was set at 15 min, and tablets prepared by adsorbing Formulation F3 were tested for their disintegration time by using different levels of croscarmellose sodium. Tablets prepared with the addition of 2% disintegrant did not disintegrate completely in 15 min, while the addition of 3% disintegrant resulted in complete disintegration. To be in the safer side by considering that the type and the concentration of lipids and surfactants used may influence the disintegration time, a 5% disintegrant level was selected for initial testing of drug release from tablets. However, as shown in Fig. 4, the drug dispersion from formulations with 5% disintegrant was incomplete despite the full disintegration of tablets, and the complete dispersion could be achieved only when the paddle speed of the dissolution apparatus was increased from 50 to 250 RPM at 3 h. In contrast, over 80% of drug dispersed in 30 min when the disintegrant level was increased to 10% (Fig. 4). It is possible that the tablets failed to break down into primary particles at the 5% disintegrant concentration. Based on these studies, the 10% disintegrant level was selected for the final formulations. It is apparent from these studies that the dispersion time, rather than the disintegration time, is a better indicator of how much disintegrant should be used in a tablet containing liquid SEDDS.

### Dispersion of Liquid SEDDS

**Fig. 5:**
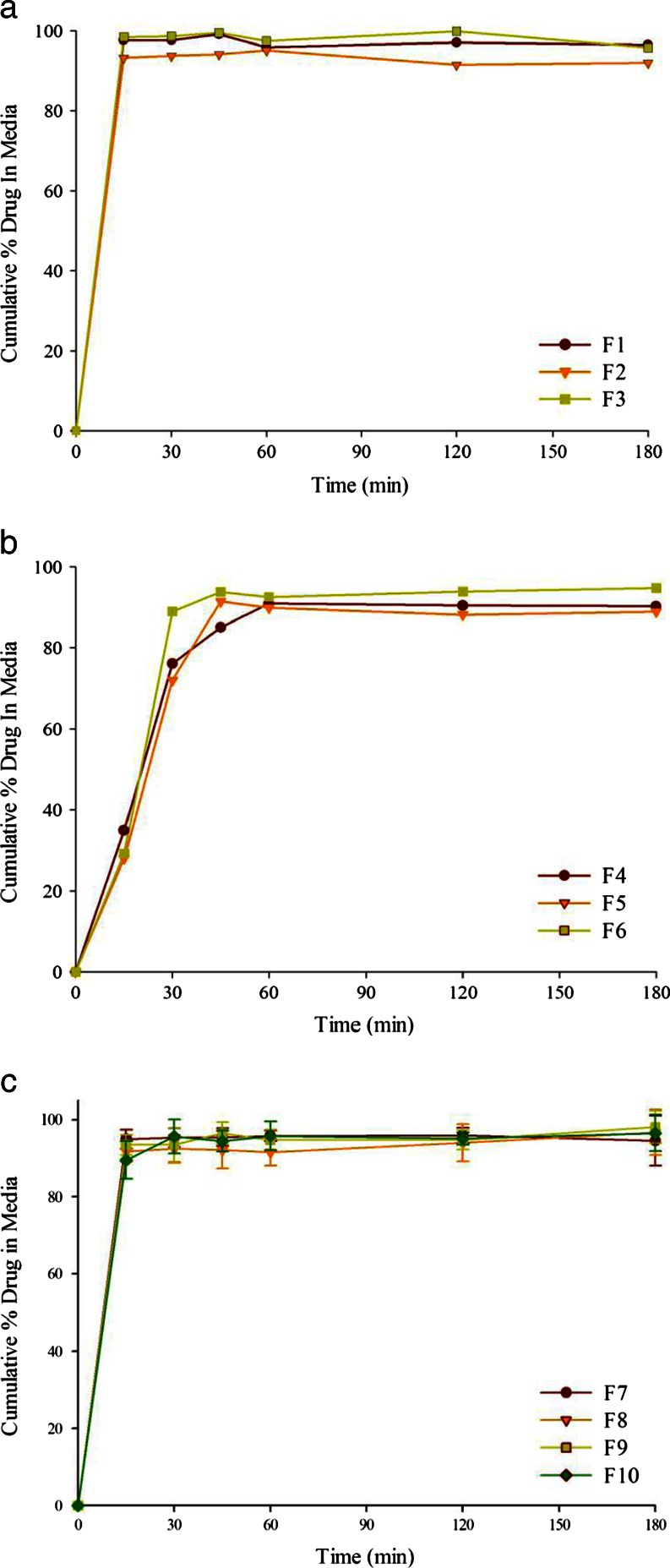
Dispersion of probucol from liquid SEDDS formulations (control group) at various ratios of (**a**) glyceryl monocaprylocaprate and PEG-35 castor oil, 7:3 (F1), 1:1 (F2), and 3:7 (F3); (**b**) caprylic/capric triglycerides and PEG-35 castor oil 7:3 (F4), 1:1 (F5), and 3:7 (F6); (**c**) glyceryl monocaprylocaprate -caprylic/capric triglycerides mixture (equal parts) and PEG-35 Castor oil 7:3 (F7), 1:1 (F8), and 3:7 (F9). Dispersion of drug dissolved in neat PEG-35 castor oil (F10) is also shown in (**c**).

The dispersion test of various formulations described in Table 1 was conducted prior to their loading onto Neusilin® US2 to determine the rate of dispersion and the particle size of oil globules produced. The concentration of probucol in the unfiltered dispersion medium at different time intervals was considered to be the measure of the extent of dispersion of the formulations. The results thus obtained would serve as reference for comparison with the dispersion of tablets prepared by adsorbing the same formulations onto Neusilin® US2. Figure 5 gives the dispersion profiles of formulations F1 to F10. No dispersion test was conducted for Formulations F11-F13, since they showed phase separation of lipids in presence of water as they did not contain any surfactants. Dispersion of Formulations F1 to F3 (Fig. 5a) and F7 to F10 (Fig. 5c) were complete in 15 min, while F4 to F6 dispersed more slowly in ~30 min. As reported earlier (30), the lipid-surfactant mixtures used in F3, F4 and F5 have the tendency to form gels in contact with water. Such an effect was responsible for the slow dispersion of the formulations, where they initially formed gels at the bottom of dispersion vessels and then dispersed slowly into microemulsions. Unlike F4, F5 and F6, the compositions of all other SEDDS (F1 to F3 and F7 to F9) formulations did not form gels and, therefore, dispersed immediately. It may be mentioned here that Cremophor EL by itself also has the tendency to form gel in contact with an aqueous medium. However, being a surfactant with relatively high hydrophilicity, any gel formed by its formulation (F10) dispersed relatively rapidly.

No precipitation of drug was observed during the dispersion test reported in Fig. 5, and no precipitates formed even when the dispersions were stored for 24 h, indicating that there was no supersaturation of drug. Thus, it was apparent that the drug remained dissolved in microemulsion, emulsion or micellar phases of the dispersions.

### Dispersion of Tablets

#### Drug Release from Different Formulations

**Fig. 6:**
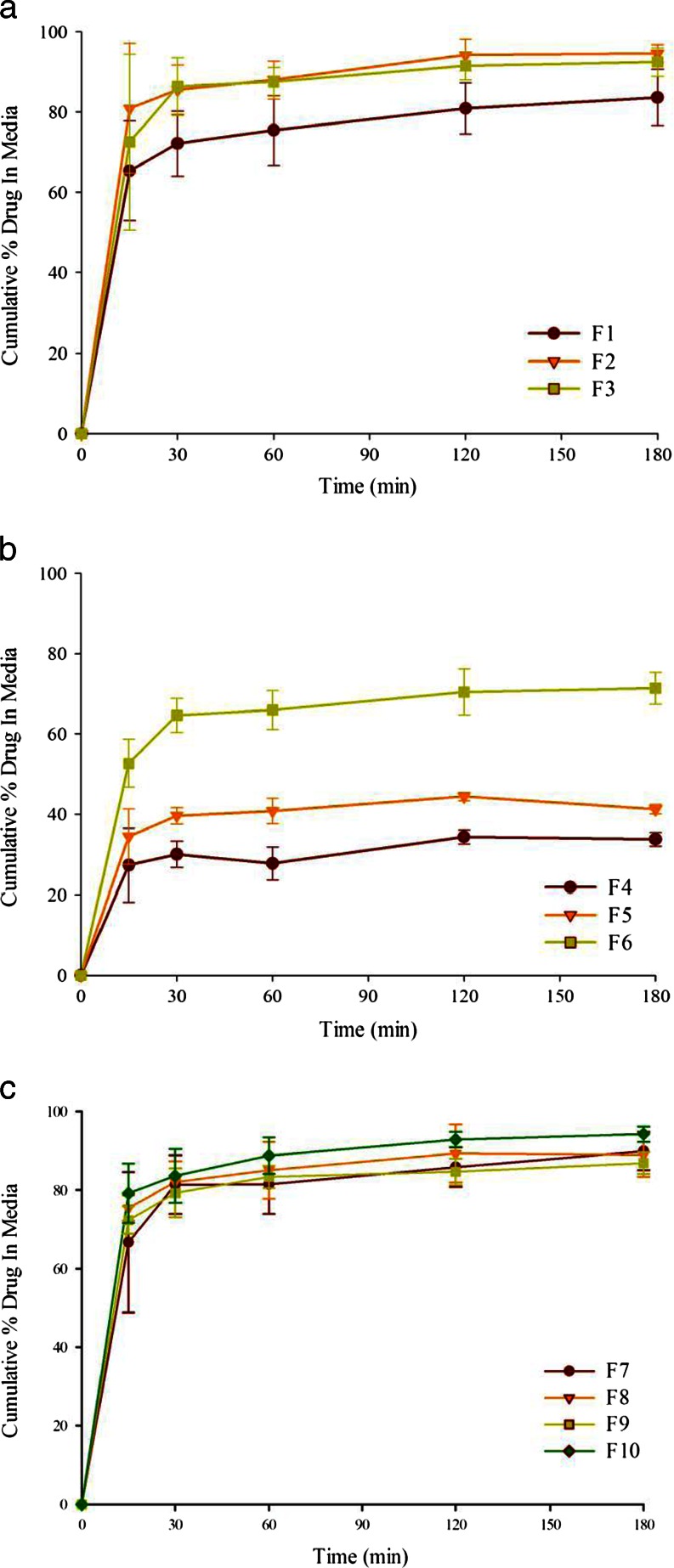
Dispersion of probucol from test group formulations (adsorbed onto Neusilin® US2) at various ratios of (**a**) glyceryl monocaprylocaprate and PEG-35 Castor oil 7:3 (F1), 1:1 (F2), 3:7 (F3); (**b**) caprylic/capric triglycerides and PEG-35 Castor oil 7:3 (F4), 1:1 (F5), 3:7 (F6); (**c**) glyceryl monocaprylocaprate (1)-caprylic/capric triglycerides (1) and PEG-35 Castor oil 7:3 (F7), 1:1 (F8), 3:7 (F9). The dispersion from the PEG-35 Castor oil (F10) formulation is also shown in Fig. 6c.

The dispersion of tablets prepared using Formulations F1 to F10 by adsorbing them onto Neusilin® US2 was conducted to investigate drug release the solid dosage form. As with the liquid formulations (Fig. 5), the drug release from the tablet formulations F1-F3 (Fig. 6a) and F7-F10 (Fig. 6c) due to the dispersion of lipids in the aqueous medium was rapid with 70–80% drug release in 15 min. The total drug release at the end of the dispersion test was ~90%, except for F1 where the dispersion profile leveled off at the drug concentration of 75–80%. The dispersion of ~90% was considered to be essentially complete as the adherence of lipids to the glass pipettes and filters used for withdrawal and filtration of aliquots could be responsible for the slight decrease drug concentration (30). In case of F1, there was a thin layer of oily liquid at the surface of the dissolution medium, possibly due to incomplete dispersion when the monoglyceride to surfactant ratio was high (7:3 w/w); the dispersion was almost 90% when the stirring rate at the end of the test was increased to 250 RPM.

Tablets prepared by using Formulations F4, F5 and F6 exhibited incomplete drug release upon dispersion in the aqueous medium. The total amounts of drug released from Formulations F4, F5 and F6 were about 35, 45 and 65% only, and the remaining drug could be accounted for by analyzing the residual solid (64, 58 and 37%, respectively). The retention of drug in the silicate was confirmed by conducting the experiments and analyzing residual solids in triplicate. It may also be noted in Fig. 5b that most of the drug was released within the first 15 min of experiment and there was very little release of the drug over the next 165 min.

#### Effect of Mixing Lipids

Figure 6c shows the dispersion profiles of Formulations F7, F8 and F9. Somewhat similar to the dispersion profiles exhibited by Formulations F2 and F3, complete drug release (>80%) was observed for all of these formulations. Since complete dispersion was observed at all lipid to surfactant ratios, the mixing of Capmul MCM EP with Captex 355 EP/NF at the 1:1 w/w ratio appeared to be more effective than using Captex 355 EP/NF alone. Moreover, the droplet size of the microemulsions formed continued to be very small (<50 nm; see further discussed in ‘Droplet Size Analysis’). These results are in agreement with the previous reports of using the mixed lipid systems (30,41).

#### Effect of Gel Formation on Extent of Drug Release

Looking at the above results, two questions arise: 1) Why didn’t Formulations F4, F5 and F6 exhibit complete drug release (Fig. 6b) similar to the control formulations (Fig. 5b), and 2) why was the drug release from Formulations F7, F8, and F9 was complete?

Formulations F4, F5 and F6 contain mixtures of caprylic/capric triglyceride (Captex 355 EP/NF) with the surfactant PEG-35 castor oil (Cremophor EL) at, respectively, 7:3, 1:1 and 3:7 w/w ratios. Previous work in our laboratory demonstrated the lipid-surfactant mixtures at these ratios form gels in contact with water (30). Although the lipid to surfactant ratios in Formulations F7, F8 and F9 were similar to those in F4, F5 and F6, a 1:1 w/w mixture of the triglyceride (Captex 355 EP/NF) and the monoglyceride (Capmul MCM EP) was used instead of using the triglyceride alone. There was no gel formation in contact with water when the mixed lipids were used (30). Thus, there is a good correlation between the gel formation and the drug release from tablet formulation containing Neusilin® US2 (Figs. 5b *vs*. 6b). Such a correlation also applies to Formulations F1, F2 and F3 where there was no gel formation and as a result the drug release was almost complete (Fig. 6a). It is postulated that the gel formation clogs the pores of Neusilin® US2, hindering the release of liquids lipid-surfactant mixtures adsorbed into the pores; only the liquids from the surface and the superficial and easily accessible parts of the pores could possibly be released. The gels formed by the formulations were tightly lodged inside the pores such that even after intense agitation at 250 RPM any additional drug release was very small (<5%).

As mentioned earlier, Formulation F10, which contained only the surfactant and there was no lipid present, also had the tendency to form a gel. By comparing it with Formulations F4, F5 and F6, a general trend may be observed in drug release from the gel-forming formulations. The higher the concentration of surfactant in the lipid-surfactant mixture, the higher was the drug release from tablets (surfactant content: F10 (100%) > F6 (70%) > F5 (50%) > F4 (30%); drug released: F10 (90%) > F6 (64%), F5 (58%) and F4 (37%). Thus, it appears that, in addition to gel formation, the increased hydrophilicity of gels, i.e., the reduction in the lipid content and the increase in the surfactant content, is a contributing factor towards increasing the drug release. Since the contrary, i.e., the increase in lipid content, is normally the intent of the development of lipid-based formulations, it is, therefore, essential that the gel formation should be avoided to ensure complete drug release from tablets containing Neusilin® US2.

In a recent report, van Speybroeck *et al*. (27) observed incomplete drug release when a lipid-based formulation essentially similar in chemical components to Formulation F7 was adsorbed onto Neusilin® US2, while no such decrease in drug release was observed when F7 was adsorbed onto the silicate. However, one important difference between the two formulations is that instead of using a 1:1 w/w mixture of Captex 355 EP/NF and Capmul MCM EP like the present investigation, van Speybroeck *et al*. used a 2:1w/w mixture. It was reported by Prajapati *et al*. (30) that a gel would be formed when the Captex 355 EP/NF to Capmul MCM EP ratio is increased above 1:1 w/w, which is the case in the reported study (27). Thus, the finding of van Speybroeck *et al*. also supports that the gel formation is responsible for the incomplete drug release from Neusilin® US2.

#### Clogging of Pores by Gel Formation

**Fig. 7:**
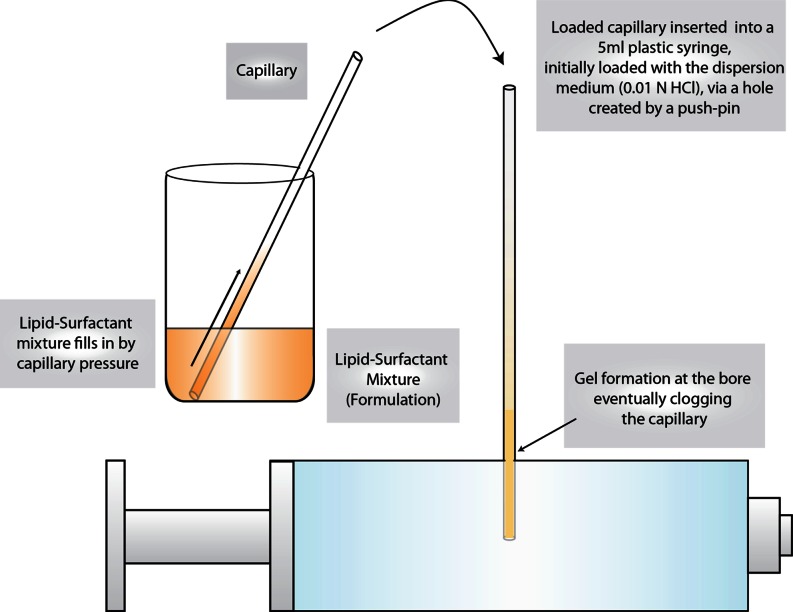
Illustration of the experimental setup to determine the effect of gel formation at the mouth of a capillary tube inserted into a syringe containing dispersion medium.

A relatively simple experiment was designed to confirm the hypothesis that the formation of gel was the principle reason behind the clogging of Neusilin® US2 pores and the incomplete drug release. The experimental design is shown in Fig. 7. A 5-mL plastic syringe was filled to about 90% of its volume with the dispersion medium (0.01 N HCl), and a capillary glass tube filled with Formulation F1, F4 or F7 was then inserted into the barrel of the syringe through a hole created with a pin. The capillary tube fitted tightly in the hole and there was no leakage of liquid from the syringe through its side. It was observed that the liquids from the capillaries containing Formulations F1 and F7 drained into the syringe with gentle shaking and mixed with the medium. On the other hand, the liquid from the capillary tube containing F4 remained within the capillary as a gel was formed at the end of the tube that came in contact with the dispersion medium. There was no drainage of Formulation F4 into the syringe even after the shaking by hand for 5 min, followed by 4 h on a wrist action, thus demonstrating that the gel formed inside the capillary was tightly lodged, blocking any passage of liquid through it.

In a modified version of the experiment, the capillary tube was inverted such that the open end faced downward. Upon gentle shaking, the liquid poured freely through the capillary tube that contained F7 but not through the tube containing F4 as it was clogged by gel formation.

**Fig. 8:**
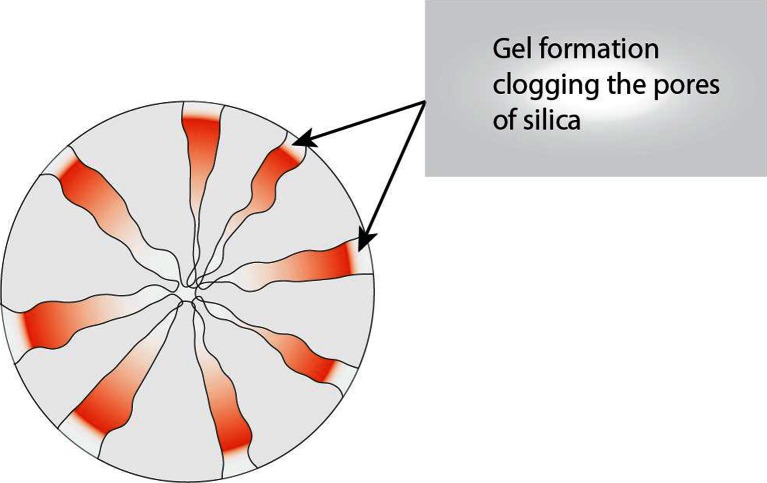
Depiction of a cross section of Neusilin®US2 with its pores filled with lipid-surfactant mixture. The red color portrays clogging of the pores due to formation gel upon contact with the dispersion medium similar to the clogging of the capillaries in Fig. 7.

As mentioned earlier, Formulations F1 and F7 do not form gels while F4 does. A schematic diagram of the gel formation clogging the pores of silicate is shown in Fig. 8. Thus, the above experiments demonstrate that the gel formation prevents the release of lipid-based drug delivery systems from silicate pores, and any gel-forming formulation should be avoided from development into tablets (or powders) by adsorbing them onto silicates.

A similar experiment was also conducted by using Formulation F10, which contained only the surfactant and no lipid was present. Although it tended to form gel within the capillary, the gel drained out within 5 min after gentle shaking by hand. Thus, the method is also capable of distinguishing between relatively hydrophobic and hydrophilic gels.

#### Particle Size Analysis

The particle size analysis after dispersion of three formulation (F7, F8 and F9) as liquids (control) and tablets in 250 mL of 0.01 N HCl are given in Table III. These formulations were chosen for particle size analysis because they were known to form microemulsions with very low particle sizes upon dilution with a dissolution medium (30). It was of interest to see whether there would be any change in particle size of microemulsions formed following the adsorption of liquids onto the silicate. Measuring the particle size of lipid-based formulations containing Neusilin® US2 was, however, very challenging due to the interference of the silicate particles. Although Neusilin® US2 has an average particle size of >60 μm, some fine particles, even in the nanometer size range, may still be present. Fine particles may also break out from the surface of Neusilin®US2 during tablet compression and the dispersion test. Because of these reasons, the data in Table III are presented as the number distribution of particle size such that any variation is easily understood. As it can be seen from the table, there is a considerable difference in the D (90%) and the ‘cumulant’ particle size for all the test and control formulations and especially for the F9 test formulation. Nonetheless, the particle size was still very low in the microemulsion range and any difference between particle sizes of the test and control formulations of F7, F8 and F9 were not substantial, thus confirming the preservation of the dispersion efficiency of liquid SEDDS even upon solidification with Neusilin® US2.

**Table III:** Number Distribution of Particle Size of Control and Test Formulations of F7, F8 and F9^a^

Formulation	Cumulant Result (nm)	D (10%) (nm)	D (50%) (nm)	D (90%) (nm)	Polydispersity Index
F7 Control^b^	49	25	31	43	0.028
F7 Test^c^	64 ± 11	23 ± 5	28 ± 6	41 ± 8	0.195
F8 Control	35	9	10	15	0.238
F8 Test^c^	59 ± 13	14 ± 2	17 ± 3	24 ± 4	0.269
F9 Control	55	11	13	19	0.203
F9 Test^c^	160 ± 28	10 ± 3	12 ± 4	27 ± 5	0.265

^a^D (10%) is the diameter of the particle below which 10% population lie, D (50%) is the diameter of the particle below which 50% of the population lie and D (90%) is the diameter of the particle below which 90% of the population lie

^b^Liquid formulation

^c^Tablets prepared by adsobing liquids (*n* = 3)

#### Stability Considerations

All dispersion tests in the present investigation were conducted within a week of the preparation of formulations. It has, however, been reported in the literature that the lipid-based systems are prone to chemical degradation of lipids and surfactants, including hydrolysis and peroxidation, especially in the case of unsaturated lipids or surfactants (12). As a result, almost all of the marketed formulations involve the use of antioxidants or special packaging and storage conditions to maintain stability (1). The potential chemical instability of lipids and surfactants upon their adsorption onto silicates is of special concern as they are spread on a large surface area, which may facilitate oxidation. The silicates may also contain metallic impurities that might catalyze chemical degradation of lipids and surfactants. In addition, drugs may also undergo chemical degradation in presence of lipids and related excipients (42), which may further deteriorate when silicates are added to the formulations. No systematic study on the effect of any potential chemical degradation of lipid-based formulations on drug release from solid SEDDS has been reported in the literature. Some initial studies in our laboratory indicated that the drug release may reduce upon longer term storage of formulations containing Neusilin®US2. No chemical degradation of drug used was, however, observed. Further studies on the longer-term chemical stability of lipids and surfactants adsorbed onto silicates are currently underway in our laboratory to explore the possible mechanism of the reduction in drug release with time. The possibility of any other mechanism of the reduction in drug release will also be investigated.

## CONCLUSION

A relatively simple organic solvent-free method for loading liquid SEDDS into powders by adsorbing them onto the silicate Neusilin®US2 has been developed. Measurement of bulk density, tap density, compressibility index, Hausner ratio and angle of repose for the 1:1 liquid to silicate ratio demonstrated acceptable flow properties for the development of the powders into a solid dosage form, e.g., tablet. There was no significant difference in the powder properties when different formulations consisting of monoglyceride (Capmul MCM EP), triglyceride (Captex 355 EP/NF) and surfactant (Cremophor EL) individually or their mixtures at different ratios were used. Although the formulations were prepared by the simple mixing of oily liquids with powders, the content uniformity of the powders were acceptable for the development into tablets. Due to its unique chemical and physical properties, Neusilin®US2 produced tablets with good tensile strength (>1 MPa) at a relatively wide range of compression pressure (45 to 135 MPa).

It was reported in the literature that certain SEDDS have the tendency to form gel when they come in contact with an aqueous medium (30,33,41). It has been established in the present investigation that such a gel formation leads to incomplete drug release from tablets (or powders) since the gel clogs pores of the silicate, thus trapping the liquid inside. On the contrary, SEDDS adsorbed onto the silicate would be completely released or dispersed if they do not form gels. The relative hydrophilicity of the gels also appears to be a contributing factor to the release of drugs from tablets; the higher the surfactant content, the higher was the drug release. It was observed that the disintegrant level in a tablet formulation needs to be carefully optimized to ensure complete dispersion of SEDDS from tablets. Based on the results of the present investigation, it is expected that SEDDS can be successfully developed into tablets.

## Electronic Supplementary Material

Below is the link to the electronic supplementary material.

ESM 1 (DOC 3623 kb)
