# Supplementary material for: Development of Solid SEDDS, V: Compaction and Drug Release Properties of Tablets Prepared by Adsorbing Lipid-Based Formulations onto Neusilin® US2
Source: Pharm Res. 2013 Jun 25;30(12):3186–99. doi: 10.1007/s11095-013-1106-4 (PMC3841580; doi:10.1007/s11095-013-1106-4)
Supplement: Supplementary file 1 — (DOC 3623 kb) [file 11095_2013_1106_MOESM1_ESM.doc]

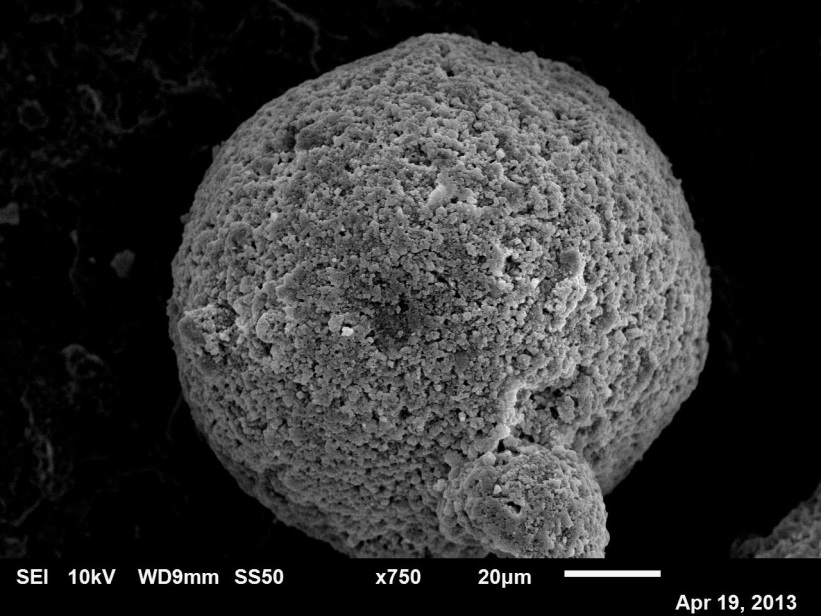

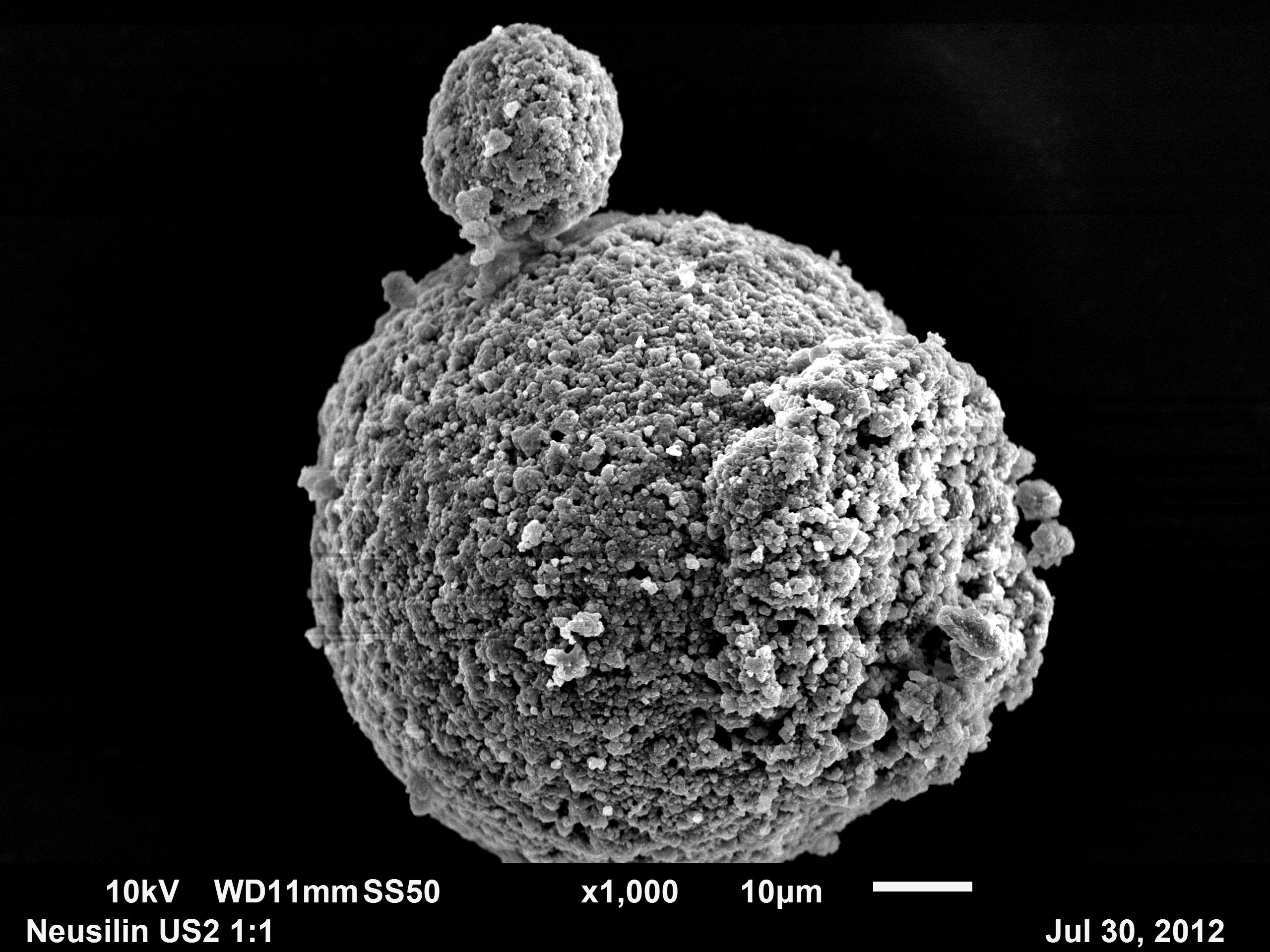

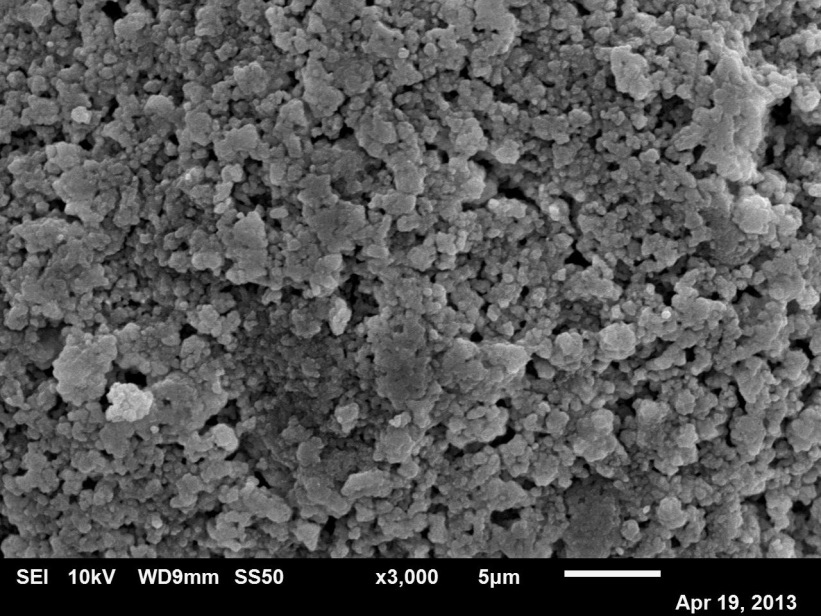

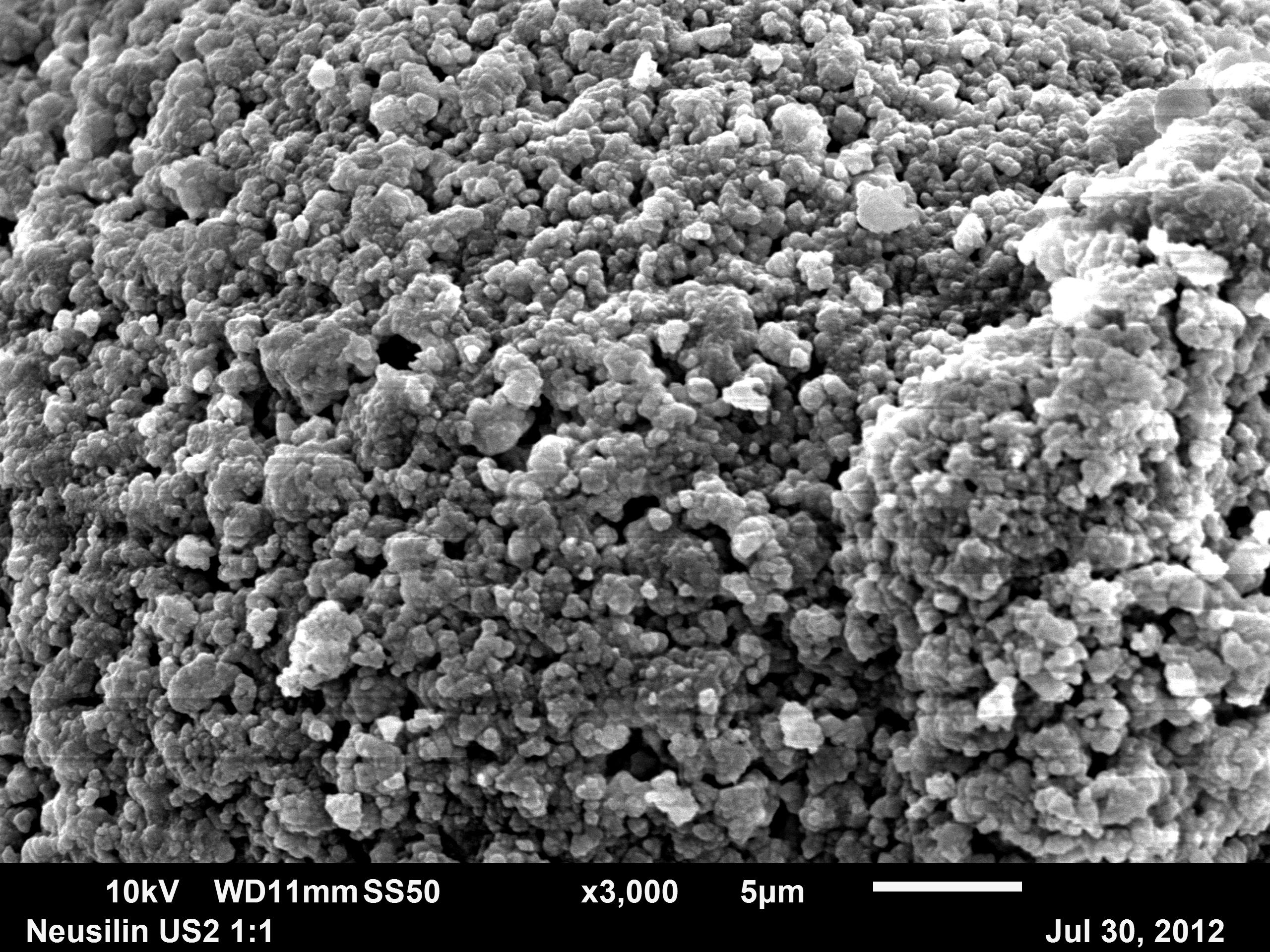

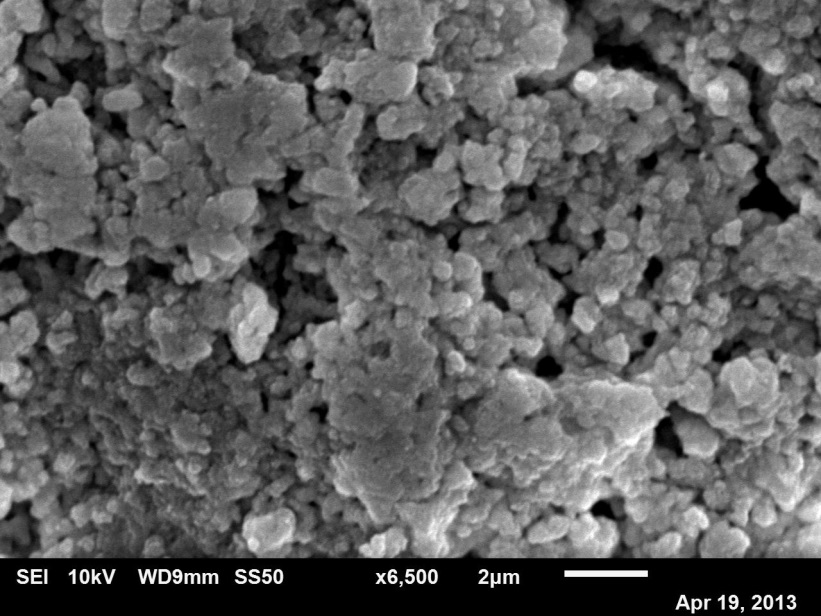

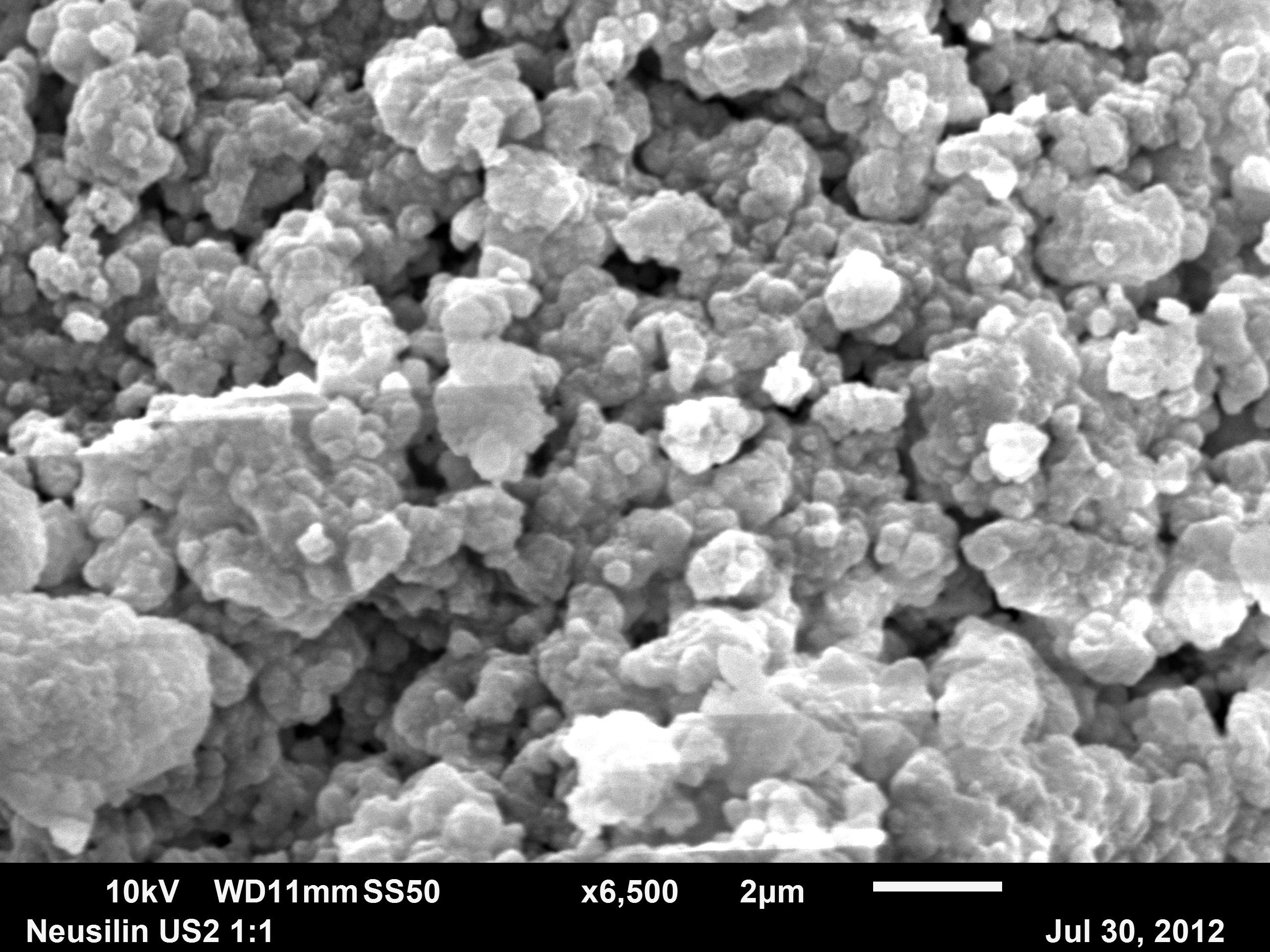


**A**

**D**

**Adsorption of Cremophor EL on Neusilin US2**

**B**

**E**

**F**

**C**

**Figure 9.**  SEM images of 1:1 w/w mixtures of Neusilin® US2 with PEG-35 castor oil (Cremophor® EL) prepared without using organic solvent at 750x (A), 3000x (B) and 6500x (C) magnifications and with using organic solvent at 1000x (D), 3000x (E) and 6500x (F) magnifications. Microscope scales are shown with the images for comparison of dimensions.

**Without Organic Solvent Used**

**With Organic Solvent Used**


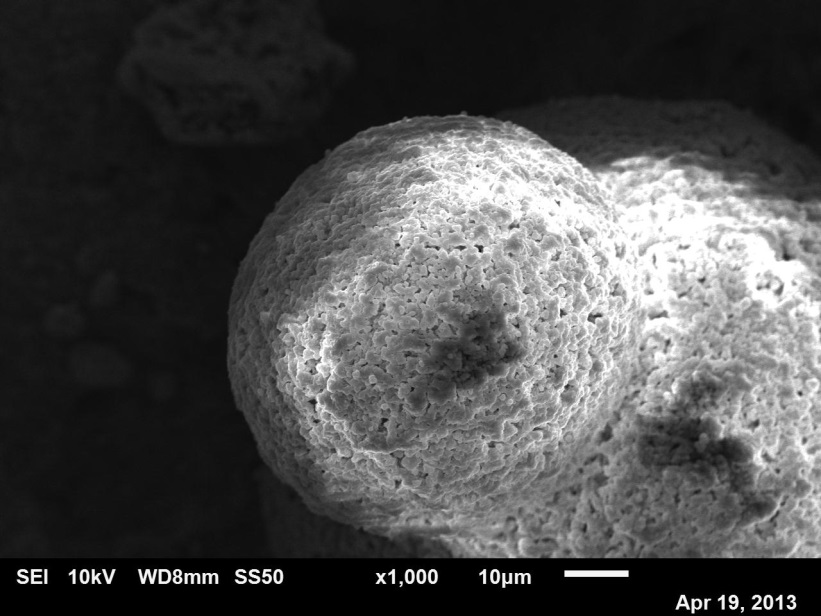

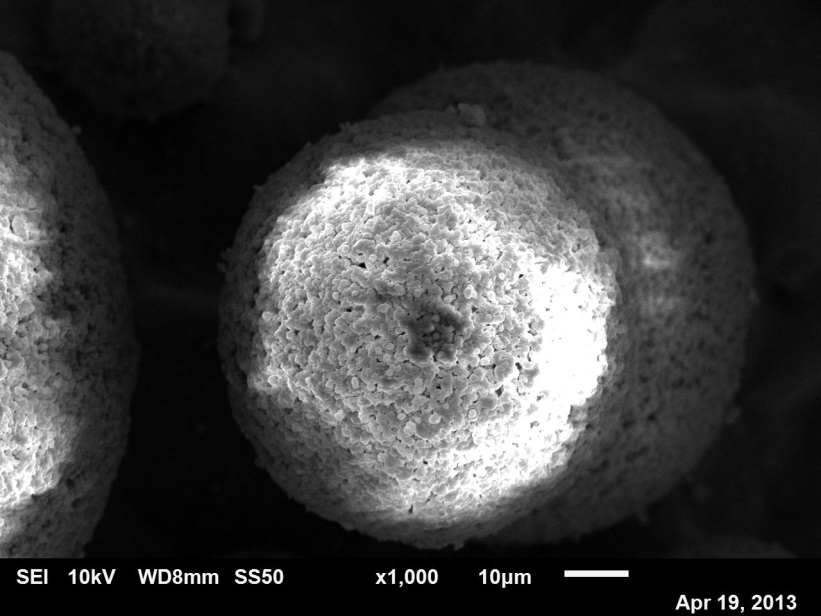

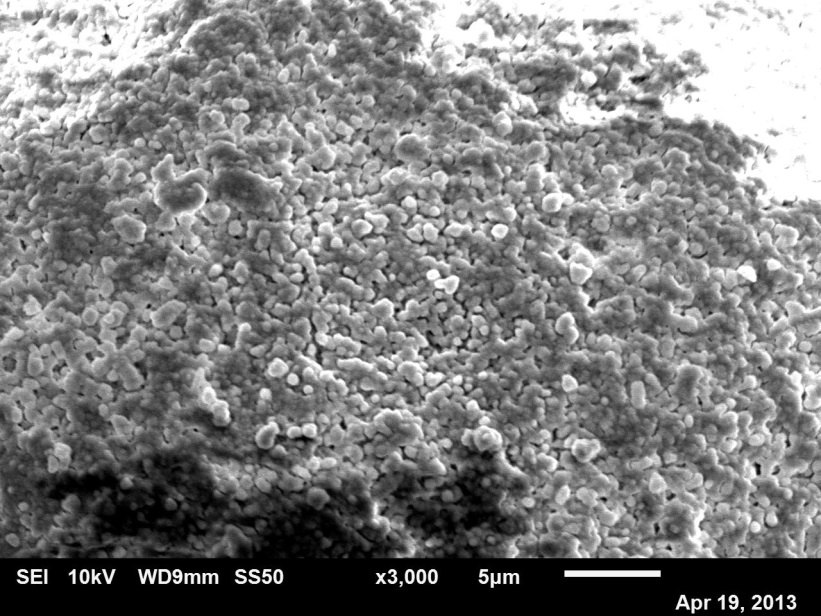

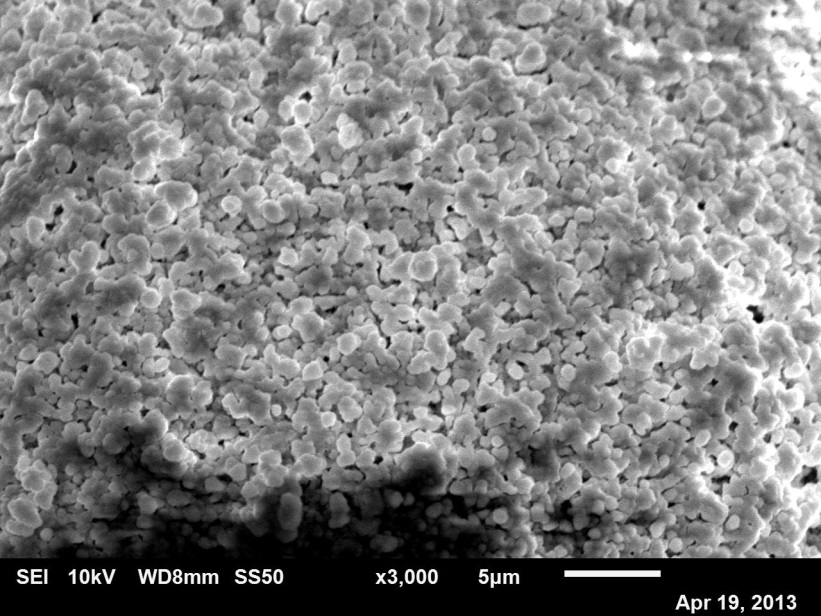

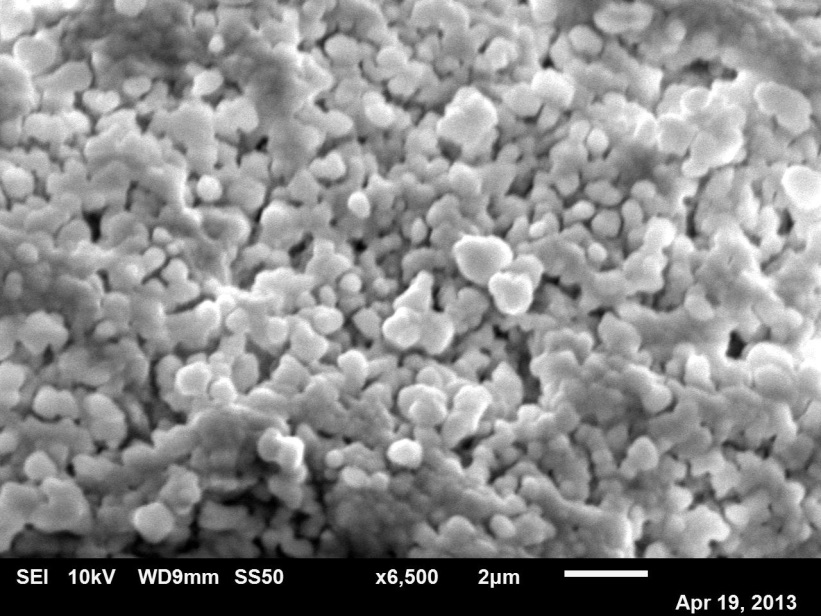

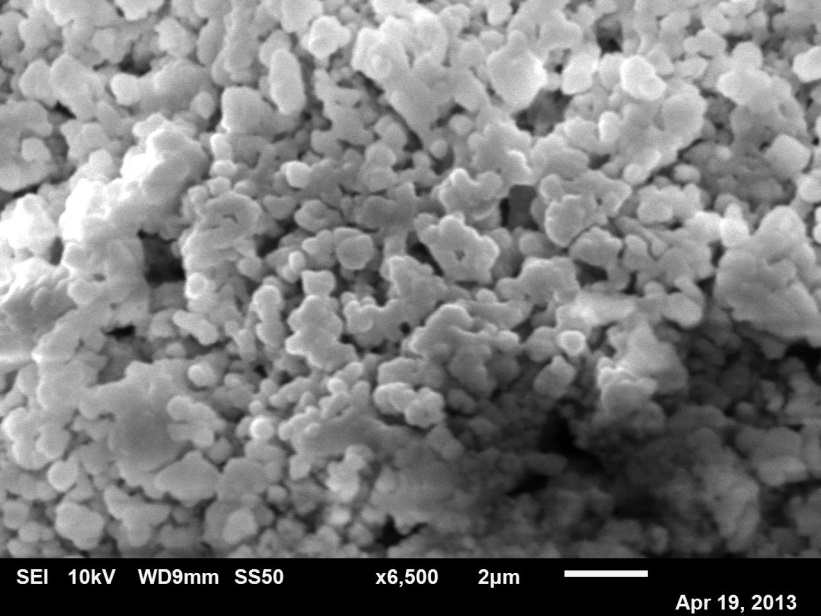


**Adsorption of Captex 355 on Neusilin US2**

**A**

**BD**

**B**

**E**

**C**

**F**

**Figure 10.**  SEM images of 1:1 w/w mixtures of Neusilin® US2 with caprylic/capric triglycerides (Captex 355) prepared without using organic solvent at 1000x (A), 3000x (B) and 6500x (C) magnifications and with using organic solvent at 1000x (D), 3000x (E) and 6500x (F) magnifications. Microscope scales are shown with the images for comparison of dimensions.

**Without Organic Solvent Used**

**With Organic Solvent Used**
